# Tailor-made gene silencing of *Staphylococcus aureus* clinical isolates by CRISPR interference

**DOI:** 10.1371/journal.pone.0185987

**Published:** 2018-01-29

**Authors:** Yusuke Sato’o, Junzo Hisatsune, Liansheng Yu, Tetsushi Sakuma, Takashi Yamamoto, Motoyuki Sugai

**Affiliations:** 1 Department of Bacteriology, Hiroshima University, Graduate school of Biomedical and Health Sciences, Hiroshima, Hiroshima, Japan; 2 Department of Mathematical and Life Sciences, Hiroshima University, Graduate School of Science, Hiroshima, Hiroshima, Japan; The University of Tokyo, JAPAN

## Abstract

Preparing the genetically modified organisms have required much time and labor, making it the rate-limiting step but CRISPR/Cas9 technology appearance has changed this difficulty. Although reports on CRISPR/Cas9 technology such as genome editing and CRISPR interference (CRISPRi) in eukaryotes increased, those in prokaryotes especially in Staphylococci were limited. Thus, its potential in the bacteriology remains unexplored. This is attributed to ecological difference between eukaryotes and prokaryotes. Here, we constructed a novel CRISPRi plasmid vector, pBACi for *Staphylococcus aureus*. The transformation efficiency of *S*. *aureus* was ~10^4^ CFU/μg DNA using a vector extracted from *dcm* negative, which encoded one of DNA modification genes, *E*. *coli*. Further, pBACi was introduced into various clinical isolates including that not accepting the conventional temperature-sensitive vector. *dcas9* in the vector was expressed throughout the growth phases of *S*. *aureus* and this vector decreased various gene mRNA expressions based on the crRNA targeting sequences and altered the knockdown strains’ phenotypes. The targeted genes included various virulence and antibiotic resistant genes. Bioinformatics suggest this vector can be introduced into wide range of low-GC Gram-positive bacteria. Because this new CRISPR/Cas9-based vector can easily prepare knockdown strains, we believe the novel vector will facilitate the characterization of the function of genes from *S*. *aureus* and other Gram-positive bacteria.

## Introduction

*Staphylococcus aureus* is a member of the normal human bacterial flora but causes a variety of diseases including from mild conditions such as skin abscess to life-threatening diseases such as toxic shock syndrome [1, 2]. *S*. *aureus* clinical isolates often exhibit multi-drug resistance and the infections from these multi drug resistant *S*. *aureus* have been an issue for decades [3, 4]. Moreover, *S*. *aureus* infects not only human but also animals [5, 6]. *S*. *aureus* infects mammary glands in ruminants and causes mastitis. Also, *S*. *aureus* infects poultry and causes Staphylococcosis. These livestock animal infections damage the primary meat industry and threaten food safety. This pathogen is also known as an etiological bacterium of food poisoning known as staphylococcal food poisoning [7, 8].

The ubiquitous feature of *S*. *aureus* comes from its genetic background [9, 10, 11]. In brief, *S*. *aureus* has an ~3.0 Mbp chromosome and more than one plasmid (in many cases). The chromosome is composed of three regions: the core-genome which is shared in many *S*. *aureus* strains, the core-variable region shared by restricted lineage(s) and mobile genomic elements transferred between cells [12]. The combination of these elements has led to genetic variation and caused pathogenic evolution and adaptation to new environments. To elucidate the pathogenic and ecological features of *S*. *aureus*, not only (whole) genome sequencing but also functional genomic study is necessary. Presently, genome manipulations using a temperature-sensitive (TS) vector and transposon are the major genetic tools used [13, 14]. They have some problems and limitations such as time-consuming manipulations and requirement of stable maintenance of plasmid in target host.

Clustered Regularly Interspaced Short Palindromic Repeat (CRISPR) and CRISPR-associated nucleases (Cas) are adaptive immune system originally identified against exogenous DNA in bacteria and archaea [15]. But this system has been no longer limited in bacteria. After the first report about targetable nuclease activity with this system [16], CRISPR/Cas9 based-bioengineering has made remarkable progress [17]. This is owing to its convenience and easiness for genetic manipulation tools. Type II CRISPR/Cas9 system based-technology, which are the most used over the laboratories, are usually needed for the design of the short RNA, named as crRNA. Introduction of vectors or RNA-protein complex to organisms makes genetic modified organisms and this is easer than the previous method. However, this recent progress since 2012 did not spread to bacteriology fields, although this system was first discovered in bacteria.

In bacteria, the CRISPR/Cas9-associated genetic editing methods in limited number of bacterial species have been reported [18, 19]. This results from the difference in DNA repairing between prokaryote and eukaryotes. LigD, the responsible protein for non-homologous end joining is found in limited bacterial species such as *Mycobacterium* and *Pseudomonas* [20], usually not found in Staphylococci and others. Thus, DNA cleaving and repairing based methods are difficult in many other bacteria. In contrast, there are non-DNA cleavage based methods, such as CRISPR interference (CRISPRi). This method suppresses gene transcription and causes gene silencing. CRISPRi tools adopt the inactivated Cas9 (dCas9). Two nuclease domains in dCas9 are mutated and this protein have only DNA binding activity without DNA cutting activity. Recent reports described the vectors for prokaryotes or eukaryotes [21–27]. Those for prokaryotes are still limited to some genus of bacteria, although this tool is considered useful.

In this study, we constructed a novel CRISPRi vector, pBACi, for *S*. *aureus* and studied its ability to transform various types of clinical isolates (including a TS-vector non-acceptable clinical strain), and silence various virulence and antibiotic resistant genes.

## Materials and methods

### Bacterial strains and culture conditions

All bacterial strains are listed in Table A in S1 File. *S*. *aureus* strains were cultured in Brain heart infusion broth, Tryptic Soy broth and on Tryptic Soy agar. *E*. *coli* strains were cultured with Luria-Bertani broth (1 L containing 10g NaCl, 10g Trypticase Peptone and 5g yeast extract) or Luria-Bertani agar. All media were purchased from Becton Dickinson (Sparks, MD) and Wako Pure Chemical Industries (Osaka, Japan). If needed, ampicillin (final Conc. 100 μg/ml, Wako Pure Chemical Industries), chloramphenicol (final Conc. 10 µg/ml, Wako Pure Chemical Industries), tetracycline (final Conc. 5µg/ml, Wako Pure Chemical Industries), D-glucose (final Conc. 1% w/vol, Nacalai tesque, Kyoto, Japan) and yeast extract (final Conc. 1% w/vol, Becton Dickinson) were added.

### *E*. *coli*-*S*. *aureus* shuttle CRISPRi vector construction

**Fig 1:**
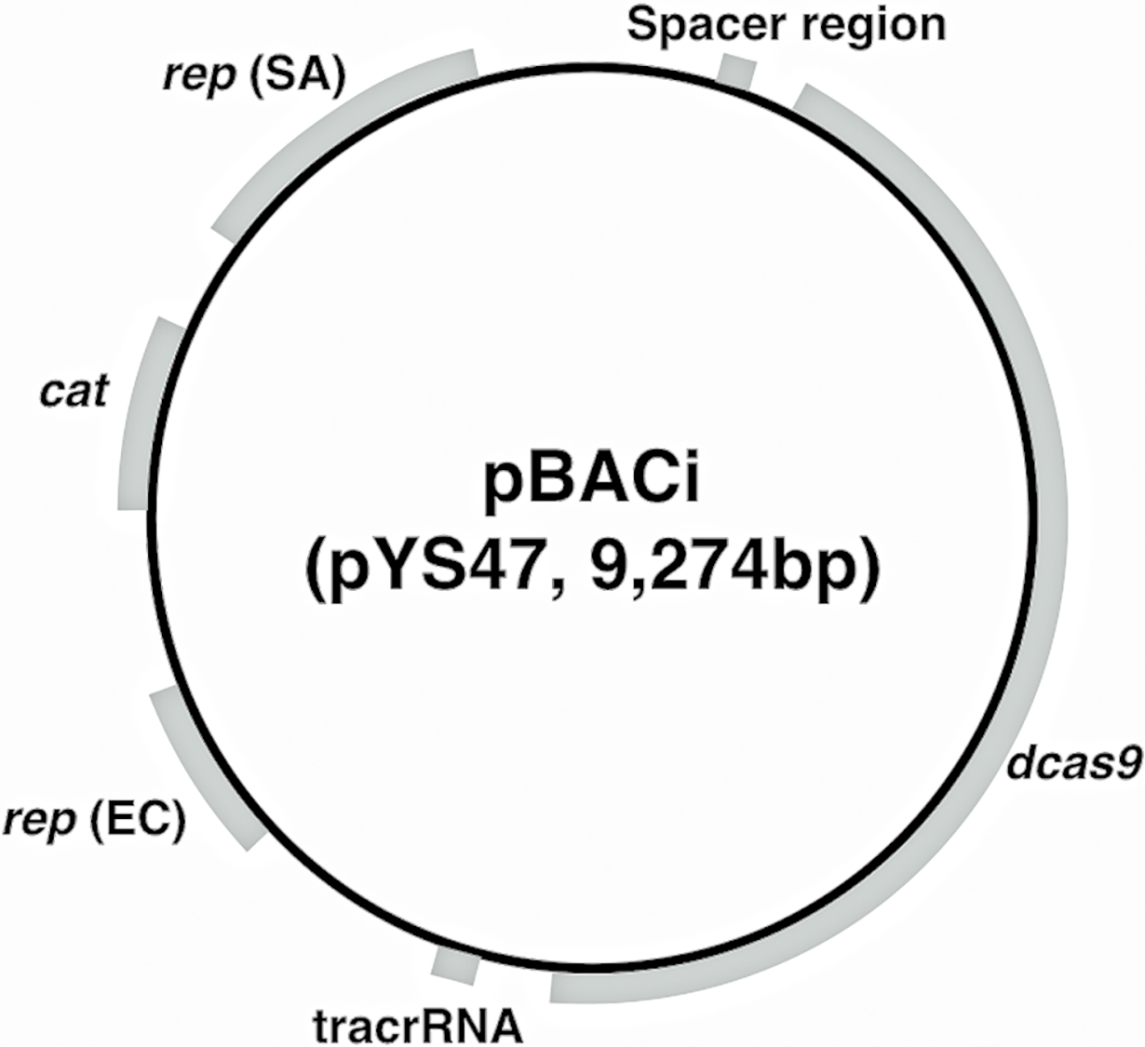
Plasmid map of pBACi, CRISPRi plasmid for *S*. *aureus*.

Plasmids and primers used for preparation of the CRISPRi vector are shown in Tables B and C in S1 File. All PCR, TA-cloning, poly-nucleotide phosphorylation and ligation were performed using PrimeSTAR^®^ GXL DNA Polymerase (Takara, Kyoto, Japan), Mighty TA-cloning Reagent Set for PrimeSTAR (Takara), T4 Polynucleotide Kinase (New England BioLabs, Beverley, MA) and Ligation high Ver. 2 (Toyobo, Osaka, Japan). *E*. *coli* DH5α was used for all cloning procedures. The construction process is shown in Fig A in S1 File. Briefly, a DNA fragment involving the CRISPR/Cas9 system component was amplified from pCas9 and cloned into pKAT. Subsequently, this intermediate plasmid was mutated by PCR-kination self-ligation. Deletion of the BsaI restriction sites and reduction of plasmid size and site directed mutations to change Cas9 to dCas9 were performed. Plasmid maps are shown in Fig 1. We named this plasmid (pYS47) pBACi. The nucleotide sequence of pBACi was confirmed with deep sequencing using Nextera XT DNA Sample Prep Kit (Illumina, San Diego, CA, USA) and Miseq (Illumina). Genome mapping was carried out with CLC Genomics Workbench (CLC Bio, Arhus, Denmark). The pBACi nucleotide sequence has been deposited in the DDBJ database under the accession code LC127310.

### Insertion of spacer sequences into pBACi and transformation into *S*. *aureus*

The primers for preparation of silencing vector are listed in Table C in S1 File and the procedure is shown on Fig B in S1 File. The vector construction was performed according to a previous method (https://www.addgene.org/42876/) with some modification. Briefly, primers were phosphorylated using T4 Polynucleotide Kinase (New England BioLabs). Then 1/25 volume 1M NaCl was added and heated at 95°C for 5 min, the primers were annealed by slowly cooling for more than 2 h. The resulting short double strand DNA was subjected to BsaI digestion (New England BioLabs) of pBACi with Ligation high Ver. 2 (Toyobo) and the ligation mixture was subsequently transformed into DH5α. After selection on Luria-Bertani agar supplemented with 10 µg/ml chloramphenicol (Wako Pure Chemical Industries), colony PCR and direct sequencing were performed with guide check S-AS primers. KOD-plus-NEO (TOYOBO) and the BigDye® Terminator v3.1 Cycle Sequencing Kit (Life technologies, Carlsbad, CA) were used in this procedure. After confirmation of the exact insertion site of the spacer sequence, the constructed plasmids were extracted with FastGene Plasmid Mini Kit (Nippon genetics, Tokyo, Japan). Extracted plasmids were subsequently transformed into *dcm*(-) *E*. *coli* B strain (BL21 or BL21(DE3)). After extraction of plasmids from the B strains, the plasmid was directly electroporated into *S*. *aureus* strains. Concentration of plasmid DNA was measured using a NanoDrop-1000 (Thermo scientific, Wilmington, DE). Preparation of *S*. *aureus* competent cells was performed as described previously [28]. Electroporation was performed using Elepo21 (NEPA GENE, Chiba, Japan) and EC-001S Nepa Electroporation Cuvettes 1 mm gap (NEPA GENE) according to the manufacturer’s protocol. The 20µl reaction mixture: 17.5µl competent cell and 2.5µl purified plasmid (10^8~9^ bacterial cells and 250-450ng plasmid DNA/reaction). Poration pulse: 1,400 V, 2.5 msec length, 50 msec interval 1 time, Polar (+). Transfer pulse: 50 V, 50 msec length, 50 msec interval, 5 times, Polar (±). All electroporation was performed at room temperature. After reaction, bacterial cells were incubated in 1 ml Brain Heart Infusion broth supplemented with 10% sucrose (Nacalai tesque) at room temperature for 60–90 min. Transformant selection was performed with using Tryptic Soy Agar supplemented with antibiotics at 37°C (pBACi or its derivative vectors) or 30°C (pKFT) overnight. PCR with Cas9 RT S-AS (or Guide check S-AS) primer sets and Quick Taq^TM^ HS DyeMix (Toyobo) were used to confirm successful transformation. Mini photo 518R (TAITEC, Tokyo, Japan, OD660) and SPECTRONIC 200 (Thermo, OD600) were used for growth assay.

### Biofilm assay

Biofilm assay was carried out using TrueLine Cell Culture Plates 96 wells (Nippon genetics). The overnight culture broth was 1,000-fold diluted with media broth supplemented with (or without) 1% glucose (Nacalai tesque) and dispensed into each well (100 µl/well). After 24 h at 37°C incubation, supernatants were discarded. After washing with 100 µl Dulbecco’s PBS (-) three times, the biofilm was stained with 1% crystal violet (Nacalai tesque) (w/vol, in water) at room temperature for 30 min. After discarding the dye solution, the plate was washed with 100 µl Dulbecco’s PBS (-) three times. The extraction of the dye was performed with 70% ethanol (Nacalai tesque) containing 1% HCl (Nacalai tesque). OD_590nm_ was measured with a Varioskan™ Flash Multimode Reader (Thermo scientific).

### Western blotting for Spa

Detection of Spa was performed using Western blot. After centrifugation of an overnight broth culture, supernatants were mixed with 2x sample buffer and incubated at 95°C for 5 min. The treated samples were used for SDS-PAGE. After electrophoresis, proteins were transferred to Hybond-P, PVDF membrane (GE Healthcare, Little Chalfont, Buckinghamshire, UK). After blocking the membrane with 5% skim milk in PBST (Dulbecco’s phosphate buffered saline with 0.05% Tween 20; Sigma-Aldrich, St Louis, MO), the membrane was incubated with 2.5 µg/ml human IgG (LLC-Cappel Products, Irvine, CA) in PBST for 1 h at room temperature or for more than 18 h at 4°C. After the washing with PBST, the membrane was incubated with 1/2,000 anti-human IgG (Goat) (LLC-Cappel Products) in PBST for 1 h at room temperature. The detection was performed with ECL Western Blot Detection Reagents (GE Healthcare) and a ChemiDoc^TM^ XRS (Bio-Rad, Hercules, CA).

### Enterotoxin detection

SEC production was assayed using SDS-PAGE and luminescent sandwich enzyme linked immuno-sorbent assay (ELISA). After centrifugation of an overnight culture, the supernatants were filtered with a Minisart (pore size 0.20 µm, Sartorius, Germany). The amount of SEC in the filtrates was assayed. For SDS-PAGE, supernatants were mixed with 2x sample buffer and incubated at 95°C for 5 min and used for SDS-PAGE. In sandwich ELISA, the samples were mixed with equal volumes of normal rabbit serum (Thermo scientific) and incubated at 4°C for 24 h. The treated samples were diluted 10–1,000 folds with Can Get Signal® Immunoreaction Enhancer Solution 1 (Toyobo) and were subjected to the assay. Preparation of recombinant SEC and SEC specific antibody preparation, and ELISA were conducted, as described previously [29–31].

### Coagulase test

Coagulase test was performed with rabbit plasma (Eiken Chemical Co., Ltd, Tokyo, Japan). After 18 h incubation at 37°C, broth cultures were centrifuged. 10µl supernatants and 500 µl plasma were mixed and incubated at 37°C. Temporal observation was performed until the control sample became coagulation.

### Beta-lactamase activity test

The β-lactamase test was performed with nitrocefin (BioVission, CA, USA). After centrifugation of an overnight broth culture, supernatants and nitrocefin solution prepared according to the manufacturer’s instruction were mixed and incubated at 37°C. Temporal observation was performed and OD_490nm_ was measured with a Varioskan™ Flash Multimode Reader.

### RNA purification, reverse transcription and quantitative PCR (qPCR)

RNA extraction was performed using a FastRNA Pro Blue Kit (Q-Biogene, Carlsbad, CA). Six hours after inoculation of about 10^8^ cells/ml (OD_660nm_ = ca. 0.1), the culture media was centrifuged. Supernatants were discarded and pellets were re-suspended in RNApro solution. Using Lysing Matrix (MP Biomedicals), RNA was extracted from bacterial cells. Remaining DNA was degraded by RQ1 RNase-Free DNase (Promega, Madison, WI). DNA-free RNA was subjected to reverse transcription (RT) with Transcriptor First Strand cDNA Synthesis Kit (Roche, Indianapolis, IN). RNA extraction, DNase treatment and RT were performed according to the manufacturer’s direction. Synthesized cDNA solution was 10-fold diluted with 10 mM Tris-HCl/1 mM EDTA buffer (pH 7.5).

The primers for qPCR are listed Table C in S1 File. The amplification efficiency of all primers was within 90–110% and PCR reaction was not inhibited. Three genes (*gyrB*, *gapDH*, *femB*) were used for reference genes. Primers for *gyrB* had been used in previous study [32], while the others were original in this study. Real time PCR and data analysis was performed as described previously [32].

### Bioinformatics analysis

We searched similar *repB* gene in DDBJ/EMBL/GenBank database. The database Version is August, 2017. Cut-off point was >80% Amino acid similarity of RepB protein.

## Results

### Construction of CRISPRi vector and transduction into clinical strains

**Fig 2:**
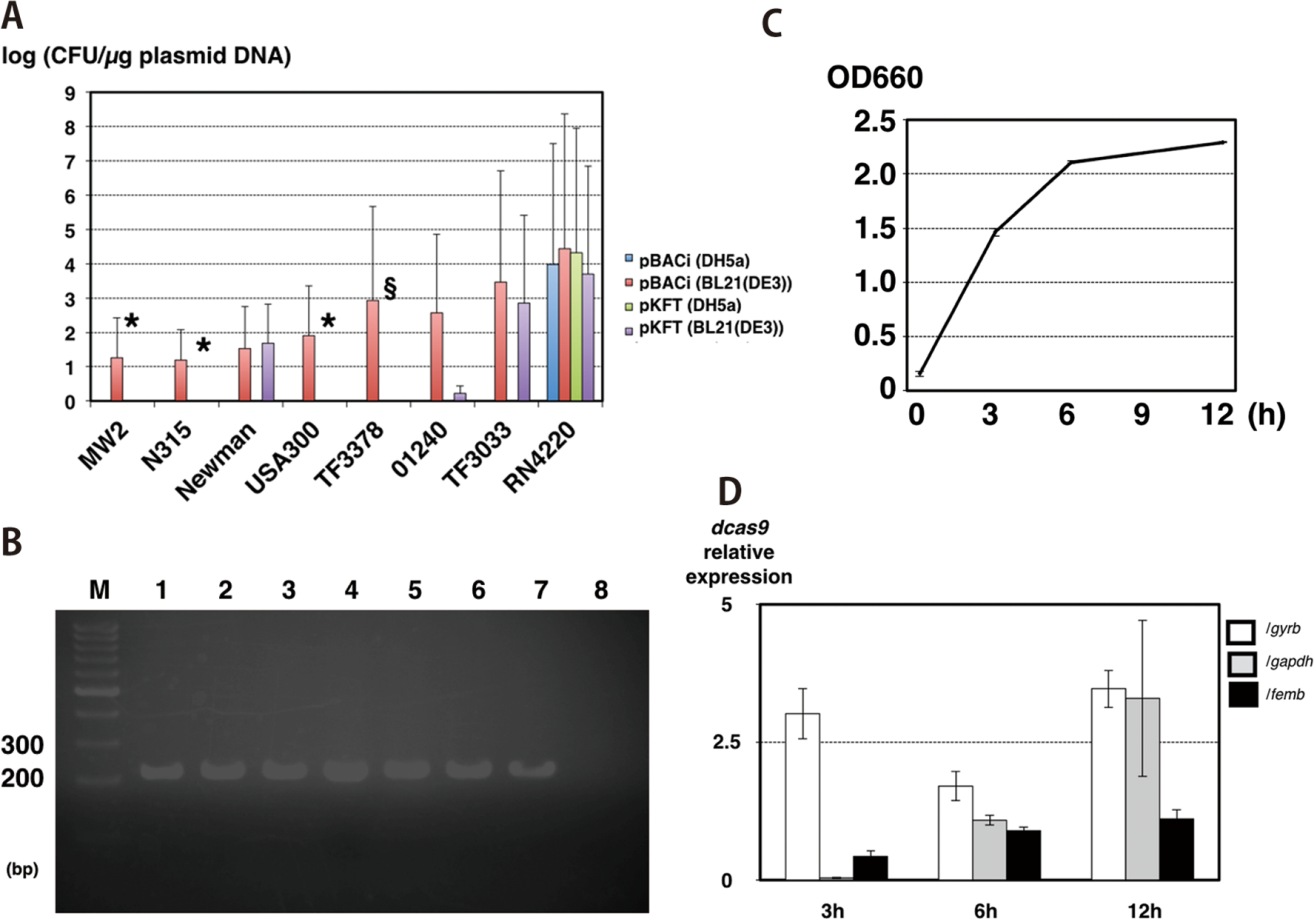
Evaluation of the novel CRISPRi plasmid for *S*. *aureus*. 2A. Transformation efficiencies of *S*. *aureus*. The transformation efficiencies of pBACi and pKFT are shown. Both plasmids were extracted from DH5α (*dcm*+) and BL21(DE3) (*dcm*-). Electroporation of all strains was performed at least three times. When no transformants were obtained until three trials, seven more electroporations (total n = 10) were conducted. *: Strains which could accept pKFT extracted from RN4220 (Transformation efficiency is 16.8~1118.2CFU/µg DNA). §: Strains which could not accept pKFT extracted from RN4220. 2B. Confirmation of correct transformation. The MW2 transformants were subjected to PCR. 1–3: Transformants using pBACi extracted from BL21(DE3). 4–6: Transformants using pBACi extracted from RN4220. 7: pBACi DNA. 8: No DNA (only purified water). M: 100bp marker. 2C. Growth curve of MW2/pBACi. Temporal measurement of OD_660_ are shown. Three independent cultures were performed. After inoculation of 1/100 volume o/n pre-culture media into fresh media (OD_660_: ~0.1), the temporal samplings (3 h: early-mid log phase, 6 h: late log phase, and 12 h: stationary phase) were performed. Vertical line: OD_660_. Horizontal line: time (hours, h). Average and standard error (SE) are shown. 2D. Relative expression of *dcas9*. The temporal expression of *dcas9* in MW2/pBACi is shown. Three genes were used as reference genes. Gyrase subunit B (*gyrB*): white, glyceraldehyde 3-phosphate dehydrogenase (*gapDH*): gray and *femB*: black. Three independent samples of three cultures and three qPCR assays were conducted (n = 9/sample). The samplings were performed at three time points as described above. Average and SE are shown.

First, we constructed CRISPRi *E*. *coli*-*S*. *aureus* shuttle vector (pBACi) with genetic engineering (Fig 1). We chose pKAT as a framework of the shuttle vectors, because we confirmed that this plasmid could be introduced into the *S*. *aureus* clinical strain, which did not accept the previous TS-vector [33]. We also used CRISPR/Cas9 components from pCas9 [19]. We estimated that these components would work well, because these were originated from *Streptococcus pyogenes*, which are also classified into low Gram-positive cocci, similar to *S*. *aureus*. We used these two major components, performed fine-tuning curetted unnecessary sequences of the plasmid, and got the final vector named as pBACi. The information on the final plasmid is shown in Table D in S1 File. This plasmid contains genes for replication in *S*. *aureus* and *E*. *coli*, an antibiotic resistance marker (chloramphenicol), *dcas9* and sequences harboring crRNA/tracrRNA for CRISPRi. We confirmed if this vector successfully transformed into *S*. *aureus* and silenced gene expressions. At first, we tried to electronically transform various clinically isolated *S*. *aureus* strains with either pBACi carrying *repB* (non-temperature sensitive) or pKFT carrying temperature-sensitive *rep* (conventional temperature-sensitive vector). *S*. *aureus* RN4220, which is restriction-negative modification-proficient and is frequently used as an intermediate strain for the transfer of plasmid DNA into the target strain, was used as a control recipient. The transformation efficiency of pBACi and pKFT is shown in Fig 2A. We tried transformation with plasmid DNA extracted from different *E*. *coli* types (*dcm* positive/negative) since *dcm* is one of DNA modification genes and we expected this gene would influence the transformation efficiency. Transformation of *S*. *aureus* with both vectors extracted from DH5α (*dcm*+) was only observed in RN4220 but not in the others. For pKFT, we obtained transformants from four strains with the vector from BL21(DE3) (Fig 2A, violet), and from three strains with vector DNA from RN4220 at 16.8~1118.2CFU/µg. However, we failed to obtain pKFT-transformed TF3378 even with DNA from RN4220 as an intermediate. In contrast, transformation with pBACi extracted from BL21(DE3) (*dcm*-) was successfully achieved in all strains including TF3378 without using RN4220 (Fig 2A, red)). We also confirmed if this transfomants possessed pBACi and these strains were not antibiotic tolerant colony. To confirm the transformation with pBACi, three independent colonies of direct transformants from BL21(DE3) (*dcm*-) and those bypassed through RN4220 were proved for the presence of pBACi. As shown in Fig 2B, pBACi was successfully transformed into MW2, showing the amplification of the pBACi specific PCR product. Also, the vector was successfully introduced into other strains (Fig D in S1 File). We confirmed *dcas9* was expressed throughout the growth phases including the mid-exponential phase (3 h), late-exponential phase (6 h) and stationary phase (12 h) as shown in Fig 2C and 2D. This demonstrates pBACi successfully transformed previously un-transformable clinical strains as well as transformable clinical strains; and *dcas9* mRNA was constantly expressed in *S*. *aureus*. We analyzed the distribution of *repB* and found that many Gram-positive bacteria possessed this gene (Table F in S1 File).

### Silencing of cell-wall protein in various strains

**Fig 3:**
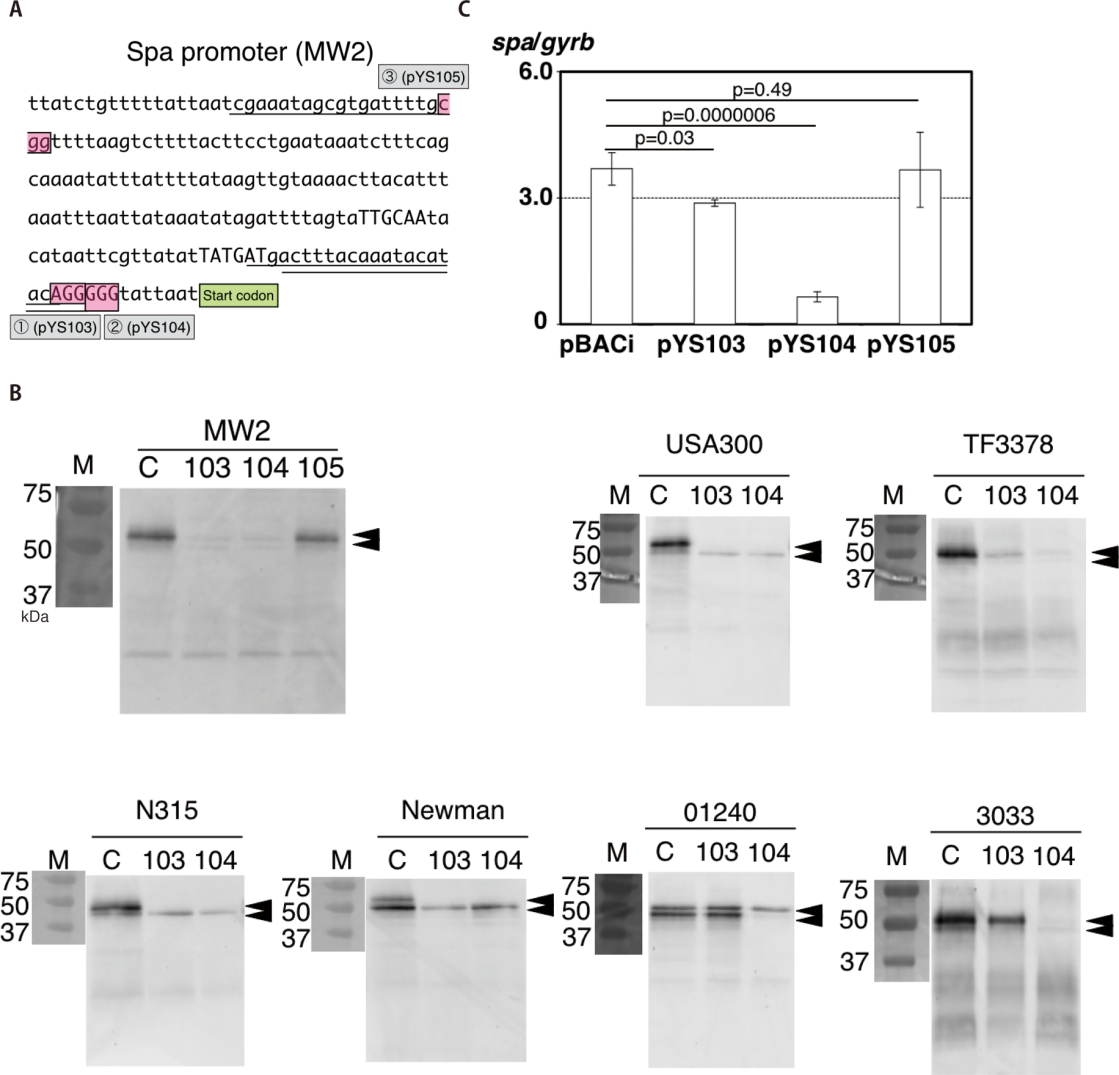
Staphylococcal protein A (Spa) silenced using CRISPRi. No significant growth inhibition was observed between the control and the silenced knockdown strains. pBACi (C): Vector control, pYS103 [103]: *spa* silenced knockdown vector 1, pYS104 [104]: *spa* silenced knockdown vector 2, pYS105 [105]: *spa* silenced knockdown vector 3. 3A. The upstream sequence of *spa* in MW2 and nucleotide sequences corresponding to spacer sequences. The partial sequence between *spa* (MW0084) and *sarS* (MW0085) in MW2 (Accession number: BA000033) is shown (101,008 bp-101,207 bp in MW2). Underline: spacer sequence [1–3], red boxes: PAM sequence (NGG), start codon: start codon of *spa* (ttg), capital letters: putative -35b, -10b and SD sequence (ribosome binding site) of *spa* in MW2. 3B. Western blotting to detect Spa in vector controls and silenced knockdown strains. Seven strains were analyzed. Three independent cultures from a single colony were performed and representative data are shown. Brackets indicate Protein A. Due to posttranslational processing of the cell surface-exposed ProteinA, some strains show double bands. “Plasmid No.” indicates serial pYS number plasmid used in this study (see S1 File). M: molecular marker. 75, 50 and 37 mean 75kDa, 50kDa and 37kDa bands, respectively. C: pBACi control vector (No crRNA coding region for *spa* silencing), pYS103-105: silencing CRISPRi vector containing the nucleotide sequence 1–3 corresponding to Fig 3A. Arrow heads: Spa. Two bands were found. This might result from processed/non-processed bands [52]. 3C. Repression of *spa* mRNA in MW2 using CRISPRi. Relative *spa* gene expression (/*gyrB*) is shown. MW2/pBACi, MW2/pYS103, MW2/pYS104 and MW2/pYS105 were independently cultured three times. Three independent qPCR were performed (n = 9/strain). The average and SE are shown. Statistics: Student’s *t*-test.

We next attempted to repress expression of virulence genes and to change phenotypes in various strains. We thought functional dCas9 protein and RNAs (crRNA and tracrRNA) were transcribed/translated and processed in *S*. *aureus*, and silenced genes, because dCas9 and crRNA/tracrRNA promoters were originated from *Streptococcus pyogenes*, which is also Gram-positive-coccus [19]. We chose *spa* gene encoding staphylococcal protein A, one of cell wall anchored proteins (Table E in S1 File). Using three crRNA recognizing sequences (Fig 3A), we developed three different pBACi (pY103, pY104 and pY105) (Fig 3A) and evaluated if and how much pBACi repressed the *spa* gene expression. The procedure for the silencing plasmid construction is shown in Fig B in S1 File. No significant growth inhibition in all knockdown strains and no significant difference with SDS-PAGE was observed (Fig E and Fig F in S1 File). As shown in Fig 3B (Upper left), pYS103 and pYS104 significantly reduced Spa synthesis, while pYS105 did not. Similarly, *spa* mRNA expressions of pYS103/MW2 and pYS104/MW2 were significantly decreased and pYS104 was more effective in repression than pYS103 (Fig 3C). In contrast, no decline in *spa* mRNA was observed using pYS105. The crRNA sequences of pYS103 and pYS104 were designed for upstream sequences close to the start codon, while pYS105 was far from the start codon (Fig 3A). Although we found almost complete inhibition of Protein A expression in both cases, repression level of mRNA by pYS103 and by pYS104 are strikingly different (Fig 3C and 3B). We do not have concrete biochemical explanation for this, but it should be noted that the timing to measure mRNA and that to measure surface Protein A is different. Post-translational proteolytic processing of Protein A exposed to the cell surface might have obscured the difference of protein expressions in two cases. We tried if the same plasmids inhibited Spa synthesis in various types of clinical strains (Table A in S1 File). As shown in Fig 3B, pYS104, the most effectively functional crRNA in MW2, inhibited Spa biosynthesis in all strains tested. Conversely, pYS103 inhibited five of seven strains and had no (or slight) effect on the other two strains, TF3033 and 01240. The crRNA recognition sequences of all tested strains were the same, as shown in Fig C in S1 File. pBACi containing the crRNA sequence corresponding to upstream sequence of the target gene clearly interfered with gene expression in *S*. *aureus* at mRNA level and altered the phenotype. Of note, design of the crRNA is assumed to be crucial in using this method. Only specified crRNA can successfully repress the same gene, *spa* gene, in wide-range strains.

### Silencing of various virulence and antibiotic resistant factors in *S*. *aureus*

**Fig 4:**
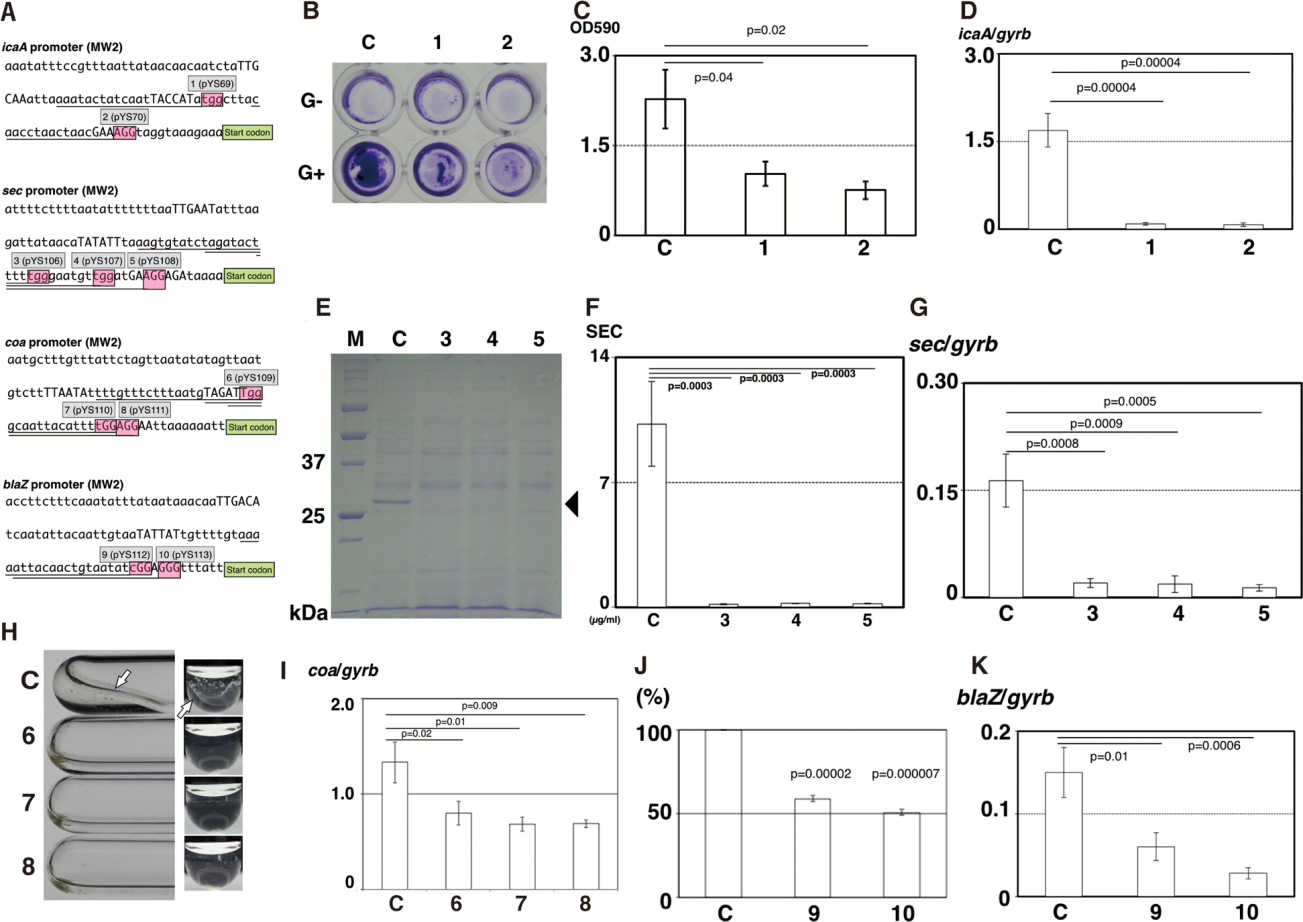
Repression of various virulence and antibiotic resistant factors in MW2 using CRISPRi. Knockdown strain names were as follows. C: MW2/pBACi (Vector control), 1. MW2/pYS69 (*icaA* silenced knockdown strain 1), 2: MW2/pYS70 (*icaA* silenced knockdown strain 2), 3: MW2/pYS106 (*sec* silenced knockdown strain 1), 4: MW2/pYS107 (*sec* silenced knockdown strain 2), 5: MW2/pYS108 (*sec* silenced knockdown strain 3), 6: MW2/pYS109 (*coa* silenced knockdown strain 1) 7: MW2/pYS110 (*coa* silenced knockdown strain 2), 8: MW2/pYS111 (*coa* silenced knockdown strain 3), 9: MW2/pYS112 (*blaZ* silenced knockdown strain 1), and 10: MW2/pYS113 (*blaZ* silenced knockdown strain 2). All phenotype assays were repeated at least three times (qPCR and ELISA: three independent cultures and three independent measurements. Other assays: three independent assays). Average and SE of each assay are shown in graphs. Statistics: Student’s *t*-test. 4A. crRNA binding regions. 100bp upstream sequences of four genes in MW2 and nucleotide sequences corresponding to spacer sequences designed in this study. Underline: spacer sequences, red boxes: PAM sequence (NGG), start codon: start codon of gene, capital letters: putative -35b, -10b and ribosome binding sites of genes. 4B. Biofilm formation on plastic surfaces. After 24 h culture, the amount of biofilm on surface was assayed. G-: without additional glucose, G+: with additional glucose. 4C. OD_590_ value of extracts from G+ wells as shown in Fig 4B. 4D. Repression of *icaA* mRNA. 4E. SDS-PAGE of supernatants. Culture media were subjected to electrophoresis. Arrow head: Protein corresponding to SEC (SEC from MW2: 28kDa). M: molecular weight marker (kDa), 4F: Sandwich ELISA of supernatants. SEC production was quantified. 4G. Repression of *sec* mRNA. 4H. Coagulase test. White arrow indicates a fibrin clot. 4I. Repression of *coa* mRNA. 4J. Measurement of β-lactamase activity. OD_490_ of nitrocephin degraded product was measured. The value of control was compared with silencing vectors (%, vertical line). 4K. Repression of *blaZ* mRNA.

We attempted to silence various genes in *S*. *aureus*. The silenced target genes, strains and plasmids are listed Tables E, A and B in S1 File, respectively. The crRNA recognition sequences and protospacer adjacent motif (PAM) sequence for each gene are shown in Fig 4A. In this study, we selected four virulence and antibiotics resistant genes: *icaA*, *sec*, *coa* and *blaZ*. No significant growth inhibition in all knockdown strains was observed (Fig E in S1 File).

*icaA* is associated with biofilm formation. As shown in Fig 4B and 4C, the mass of the biofilm formed on plastic surface in the silenced knockdown strains (1 and 2) were significantly lower than that in the control strain (C). Along with phenotypic change, *icaA* mRNA was significantly decreased in the knockdown strains (Fig 4D). Next targeted gene, *sec* encodes staphylococcal enterotoxin type C (SEC), one of enterotoxin family genes, secreted into the culture broth. As shown in Fig 4E, the ~30 kDa protein band (mature SEC in MW2: 28kDa) disappeared in the culture broth of all silenced knockdown strains (3–5). Sandwich ELISA confirmed that the amount of SEC in the silenced knockdown strains (3–5) was 50–100 fold lower than the control strain (C) (Fig 4F). This protein decreased along with *sec* mRNA repression (Fig 4G). *coa* encodes the coagulase, converting fibrinogen to fibrin. As shown in Fig 4H, all three mutants (6–8) suppressed coagulation of normal rabbit plasma, compared with the control (C). This was caused by *coa* mRNA suppression (Fig 4I). The *blaZ* encodes β-lactamase associated with antibiotic (β-lactam) resistance. Nitrocefin has a β-lactam ring where cleavage of this ring in the substrate leads color change from yellow to red. As shown in Fig 4J, β-lactamase activity in the silencing vectors (9 and 10) was reduced by about 50%. This also occurred in together with the inhibition of mRNA transcription (Fig 4K).

## Discussion

Here, we constructed a novel CRISPRi vector and evaluated its abilities. This vector was successfully introduced into various *S*. *aureus* clinical isolates with enough transformation efficiency. Also, the vector expressed CRISPR/dCas9 components and silenced gene expressions, depending on the crRNA sequences.

We found pBACi suppressed various gene expressions by changing the spacer sequence in pBACi. These genes have different characters (Table E in S1 File). *spa*, *ica* and *coa* are harbored on the chromosome; while *sec* and *blaZ* are on mobile genetic elements. Also, the location of the gene products varies: SEC, coagulase and β-lactamase are secreted outside the bacterial cell; while Spa remains on the peripheral surface and IcaA remains inside the cell. Further, the gene functions are diverse: Spa is one of the cell wall anchored proteins and functions in immune evasion binding the Fc domain of IgG and disturbing IgG recognition on the bacterial cell [34]; IcaA is one of components producing polysaccharide intercellular adhesin (PIA) in biofilm formation [35, 36]; SEC is a secreted toxin and, has emetic and superantigenic activities, causing food poisoning and toxic shock syndrome, respectively [7, 8]; coagulase is a protein that non-enzymatically activates prothrombin, converting fibrinogen into fibrin causing coagulation by forming a fibrin polymer [37]; and β-lactamase is an enzyme hydrolyzing β-lactam antibiotics into ineffective degradation products, causing resistance to β-lactams [38, 39]. As described in the results, pBACi could suppress these gene transcliptions and change phenotypes, regardless of localizations and functions of genes and proteins. Also, These phenotypes were altered by constructing a plasmid with a variety of spacer sequences and transformation (Figs 3 and 4). The same vector silenced *spa* gene expressions in a variety of strains (Fig 3B).

Our data clearly show that great care should be necessary in the design of the spacer sequence. In the case of *spa*, little space between spacer sequence (PAM) and start codon resulted in better suppression of the gene. Because the interference by pYS103 is weaker than that by pYS104, alteration of the phenotype was not significantly apparent in some strains (01240 and TF3033). Similar phenomenon was also found in other genes. *coa* mRNA repression in pYS110 and pYS111 is more effective than that in pYS109, as spacer sequences in pYS110 and pYS111 were nearer than that in pYS109. Also, in repression of *blaZ* mRNA, pYS113 was more effective than pYS112. Similar phenomenon was also observed in *E*. *coli* in a previous study [40]. But there is a little difference. The most effective crRNA for CRISPRi in *E*. *coli* was around promter (between -10b and -35b), not around SD sequence. But those in *S*. *aureus* were downstream of promoters, around SD sequence, in this study. This may probably come from the difference of bacteria species (Gram-positive or Gram-negative). We should take care this distinguishes, because the layout of the spacer sequence (crRNA) is critical for silencing. But we clearly showed that the same vector could repressed the same gene among broad strain in case of *spa*. As long as crRNA and vector is correctly designed, the same (similar) repression among varied strains may be probably adapted for other genes. Taken together, we demonstrated that pBACi can be used for production of genetically modified knockdown strains for *S*. *aureus*. Like other CRISPR/Cas9 technologies, a fine tuning of the spacer design is necessary.

There are many advantages in pBACi for knockdown strain construction platform for genomic analysis. The first is wide range of application. We confirmed that this vector could modify bacterial phenotypes, as described above. Among these phenotypes, β-lactam resistance is caused by *blaZ* harboring on a plasmid. While we demonstrated knockdown of virulence gene and antibiotics resistance gene (non-essential genes), other researchers also demonstrated essential gene knockdown in other bacteria [21, 26, 27, 41]. These tuning, switching on and off of genes are possible with the inducing regents and inducible promoters. In case of trying to knockdown essential gene(s), inducible promoters, such as tetracycline inducible promoter can be adapted to pBACi [14]. The conversion of natural promoters to inducible promoters of *dcas9* or crRNA in pBACi may allow tunable-dose expression similar to antisense knockdown system. Analyses of plasmid genes and essential genes have been hard and inefficient by the previous gene modification system using TS-vector. Thus, CRISPRi including pBACi will probably promote these studies. The second is simplicity. Previously, the TS vector method has been commonly used for the preparation of *S*. *aureus* gene-specific mutants. The TS vector contains temperature-sensitive replication genes, which do not function to maintain the plasmid at higher temperature [13, 14]. For the TS vector method, a gene specific knockout strain is selected through multiple steps including construction of a plasmid, electroporation, a series of temperature shifts, antibiotic-susceptibility test and colony screening. This takes much time (2–4 weeks) and costs a lot (enzymes, antibiotics and media). In contrast, CRISPRi method including pBACi is easy. It is very quick (within a week to construct knockdown strains) and simple (only requires integration of the spacer sequence, single electroporation and single antibiotic selection) by pBACi (Fig B in S1 File). The third is specificity. Antisense RNA method uses RNA base-paring to the target gene and reduces the target gene mRNA [14]. But, there is a risk of unexpected and undesirable base pairing to non-targeted mRNA, similar to RNAi [42]. Moreover, relatively longer counter antisense RNAs (more than hundreds base) are usually used for functional analyses of *S*. *aureus* genes [43–45] and genetic engineering [28, 46]. This enhances off-targeted mRNA degradation. In contrast, with CIRPSRi including pBACi, the gene specificity is determined by a short sequence, 20 bp spacer and 3 bp PAM suggesting more accurate performance, although the risk of off-target still remains. These are advantages of pBACi CRISPRi system. But the disadvantage of pBACi method is designation of crRNA. Sometimes, researchers should try some several types of crRNA, as described in Spa in our study. Also, polar activity of pBACi in case of polycistronic mRNAs should be paid attention.

Previous studies show many clinical strains are hard to transform with plasmid [13, 28, 47]. Likewise, we did not obtain TF3378/pKFT even by way of RN4220. Previous studies have some explanation [13, 28]. Restriction enzymes are important for the rejection of vectors. Depending on the methylation status, these enzymes digest foreign DNAs in the bacterial cell. A previous study reported inactivation of restriction enzymes increased transformation efficiency [48]. There is also a possibility that this TS-vector resistant strain did not become competent status under the growth condition in this study and other growth condition or treatments may provide the strains with competency as suggested previously [28]. Another report suggested the tropism of plasmids caused by the genetic factor(s) affecting replication or maintenance of the plasmid in another species[49]. These factors may cause low or no-transformation efficiency in present and previous studies. To overcome these issues, we developed a novel vector system to suppress the interested gene(s) bypassing the problem of long maintenance of the plasmid in *S*. *aureus*. Indeed, we succeeded in obtaining TF3378/pBACi knockdown strains at high transformation efficiency (Fig 2A).

Here, we also attempted another approach to further facilitate transformation. The restriction/modification system between *S*. *aureus* and *E*. *coli* is a barrier and direct transformation from *E*. *coli* strains such as K12 derivative strains to *S*. *aureus* clinical strains is difficult in most cases. Thus, RN4220, which is deficient in a restriction system, has been generally used as an intermediate strain for the transfer of plasmid DNA into the target *S*. *aureus* clinical strains. However, extraction of plasmid DNA from *S*. *aureus* is more complicated and time-consuming than that from *E*. *coli*. Recent reports have shown the importance of the type IV restriction system, which recognizes C^5m^ that degrades exogenous DNA [13, 50, 51] and this is the primary barrier preventing direct transformation of *S*. *aureus* with *E*. *coli* DNA. Previous studies attempted to construct a modified K12 derivative strain (*dcm-*) and subsequently achieved successful direct transformation from *E*. *coli* into *S*. *aureus* [51]. In this study, we used *E*. *coli* B strain derivatives as a plasmid donor, because these strains lack *dcm*. As shown in Fig 2A, we successfully collected pBACi transformants with vector DNA derived from the B strain. Although *E*. *coli* B strain derivatives are usually used for recombinant protein production, these strains can be also used as intermediate strains to transform plasmid vectors from *E*. *coli* cloning strains to *S*. *aureus* clinical isolates. This approach leads to further time saving using the pBACi method without preparing intermediate transformants.

In other aspects or ways, RepB in pBACi and its similar replication initiator proteins were found in plasmids from various bacterial species, most of which are classified into Bacillales (Table F in S1 File). It may be possible that pBACi can replicate and silence genes in these Gram-positive bacterial species. Thus pBACi can be adapted to not only *S*. *aureus* but also other Gram-positive bacteria, although we need to modify the vector such as the promoter sequences.

In conclusion, pBACi is a new tool to quickly knockdown gene(s) of your interest and can be expected to promote functional genomics research in *S*. *aureus*.

## Supporting information

S1 File Table A in S1 file. Table B in S1 file. Table C in S1 file. Table D in S1 file. Table E in S1 file. Table F in S1 file. Fig A in S1 file. Fig B in S1 file. Fig C in S1 file. Fig D in S1 file. Fig E in S1 file. Fig F in S1 file.
(PDF)
