# Supplementary material for: Tailor-made gene silencing of Staphylococcus aureus clinical isolates by CRISPR interference
Source: PLoS One. 2018 Jan 29;13(1):e0185987. doi: 10.1371/journal.pone.0185987 (PMC5788344; doi:10.1371/journal.pone.0185987)
Supplement: S1 File — Table A in S1 file. Table B in S1 file. Table C in S1 file. Table D in S1 file. Table E in S1 file. Table F in S1 file. Fig A in S1 file. Fig B in S1 file. Fig C in S1 file. Fig D in S1 file. Fig E in S1 file. Fig F in S1 file. (PDF) [file pone.0185987.s001.pdf]

S1 File

Table A in S1 file. Bacterial strains used in this study

S. aureus wild type strains	Clonal complex	Characteristics (Origin)	Ref.
MW2	CC1	CA-MRSA	53
N315	CC5	HA-MRSA	54
Newman	CC6	Invasive-MSSA	55
USA300	CC8	CA-MRSA	56
TF3378	CC59	Allergic skin disease	in this study
01240	CC81	Food poisoning	31
TF3033	CC509	SSSS*	in this study
RN4220	CC8	Genetic manipulation strain	57
S. aureus mutants (knockdown strains)		Silencing genes	Ref.
MW2/pBACi		- (Vector control)	in this study
MW2/pYS69		*icaA*	in this study
MW2/pYS70		*icaA*	in this study
MW2/pYS103		*spa*	in this study
MW2/pYS104		*spa*	in this study
MW2/pYS105		*spa*	in this study
MW2/pYS106		*sec*	in this study
MW2/pYS107		*sec*	in this study
MW2/pYS108		*sec*	in this study
MW2/pYS109		*coa*	in this study
MW2/pYS110		*coa*	in this study
MW2/pYS111		*coa*	in this study
MW2/pYS112		*blaZ*	in this study
MW2/pYS113		*blaZ*	in this study
N315/pBACi		-	in this study
N315/pYS103		*spa*	in this study
N315/pYS104		*spa*	in this study
Newman/pBACi		-	in this study
Newman/pYS103		*spa*	in this study
Newman/pYS104		*spa*	in this study
USA300/pBACi		-	in this study
USA300/pYS103		*spa*	in this study
USA300/pYS104		*spa*	in this study
TF3378/pBACi		-	in this study
TF3378/pYS103		*spa*	in this study
TF3378/pYS104		*spa*	in this study
01240/pBACi		-	in this study
01240/pYS103		*spa*	in this study
01240/pYS104		*spa*	in this study
TF3033/pBACi		-	in this study
TF3033/pYS103		*spa*	in this study
TF3033/pYS104		*spa*	in this study
E. coli		Characteristics	Ref.
DH5α		Construction of vectors	Takara
BL21(DE3)		Preparation of vectors for electroporation	Novagen
BL21		Preparation of vectors for electroporation	Novagen

*Staphylococcal scalded skin syndrome

Table B in S1 file. Plasmids used in this study

Plasmids	Characteristics	Reference
pKAT	*E. coli*-*S. aureus* shuttle vector	58
pCas9	CRISPR-Cas9 plasmid for *E. coli*	19
pKFT	TS-vector	59
pGEM-T easy	TA-cloning vector	Promega
pBACi (pYS47)	CRISPRi plasmid vector	in this study
pYS69	Silencing of *icaA* (type 1)	in this study
pYS70	Silencing of *icaA* (type 2)	in this study
pYS103	Silencing of *spa* (type 1)	in this study
pYS104	Silencing of *spa* (type 2)	in this study
pYS105	Silencing of *spa* (type 3)	in this study
pYS106	Silencing of *sec* (type 1)	in this study
pYS107	Silencing of *sec* (type 2)	in this study
pYS108	Silencing of *sec* (type 3)	in this study
pYS109	Silencing of *coa* (type 1)	in this study
pYS110	Silencing of *coa* (type 2)	in this study
pYS111	Silencing of *coa* (type 3)	in this study
pYS112	Silencing of *blaZ* (type 1)	in this study
pYS113	Silencing of *blaZ* (type 2)	in this study

Table C in S1 file. Primers used in this study

Primer name	Sequence (5'-3')	Characteristics
pCAS9 cas9-spacer S	gccgCTGCAGtaacaatgcgctcatcgtca	Construction of CRISPRi plasmid
pCAS9 cas9-spacer AS	gccgCTGCAGcgatggatatgttctgccaa	Construction of CRISPRi plasmid
pKAT-Cas9 modification 1AS	atacgagtgacctctcttgt	Construction of CRISPRi plasmid
pKAT-Cas9 modification 1S	cttttattcagcaatcggat	Construction of CRISPRi plasmid
pKAT-Cas9 modification 2AS	gaaaagttagaccatggaga	Construction of CRISPRi plasmid
pKAT-Cas9 modification 2S	cactttttgtcttgtccact	Construction of CRISPRi plasmid
pKAT-Cas9 modification 3S	aattgcatcaacgcatatag	Construction of CRISPRi plasmid
pKAT-Cas9 modification 3AS	ccttggcagaacatatccat	Construction of CRISPRi plasmid
Nickase primer S2	tagctatcggcacaaatagc	D10A mutation in Cas9
Nickase primer AS2	agcctattgagtatttctta	D10A mutation in Cas9
Nickase check primer S	gatactgtggcggtctgtat	Confirmation of D10A mutation
Nickase check primer AS	tttcggaggtcattaaaact	Confirmation of D10A mutation
CRISPRi primer S	gcaattgttccacaaagtttcc	H840A mutation in Nickase
CRISPRi primer AS	atcgacatcataatcactta	H840A mutation in Nickase
CRISPRi check primer S	gagagcgtatgaaacgaatc	Confirmation of H840A mutation
CRISPRi check primer AS	cttagtgatttggcgagttt	Confirmation of H840A mutation
guied RNA Check primer S	tgtggaattgtgagcggata	Confirmation of Spacer sequence
guied RNA Check primer AS	acgcattgatttgagtcagc	Confirmation of Spacer sequence
IcaA CRISPRi crRNA 1S	AAACaaatactatcaattaccataG	Spacer sequence for *ica*A type1
IcaA CRISPRi crRNA 1AS	AAAACtatggtaattgatagtattt	Spacer sequence for *ica*A type1
IcaA CRISPRi crRNA 2S	AAACcaacctaactaacgaaaggtG	Spacer sequence for *ica*A type2
IcaA CRISPRi crRNA 2AS	AAAACacctttcgttagttaggttg	Spacer sequence for *ica*A type2
SPA CRISPRi crRNA 1S	AAACatgactttacaaatacatacG	Spacer sequence for *spa* type1
SPA CRISPRi crRNA 1AS	AAAACgtatgtatttgtaaagtcat	Spacer sequence for *spa* type1
SPA CRISPRi crRNA 2S	AAACactttacaaatacatacaggG	Spacer sequence for *spa* type2
SPA CRISPRi crRNA 2AS	AAAACcctgtatgtatttgtaaagt	Spacer sequence for *spa* type2
SPA CRISPRi crRNA 3S	AAACatcgaaatagcgtgattttgG	Spacer sequence for *spa* type3
SPA CRISPRi crRNA 3AS	AAAACcaaaatcacgctatttcgat	Spacer sequence for *spa* type3
SEC CRISPRi crRNA 1S	AAACaagtgtatctagatacttttG	Spacer sequence for *sec* type1
SEC CRISPRi crRNA 1AS	AAAACaaaagtatctagatacactt	Spacer sequence for *sec* type1
SEC CRISPRi crRNA 2S	AAACtagatactttttgggaatgtG	Spacer sequence for *sec* type2
SEC CRISPRi crRNA 2AS	AAAACacattcccaaaaagtatcta	Spacer sequence for *sec* type2
SEC CRISPRi crRNA 3S	AAACtttttgggaatgttggatgaG	Spacer sequence for *sec* type3
SEC CRISPRi crRNA 3AS	AAAACtcatccaacattcccaaaaa	Spacer sequence for *sec* type3
Coa CRISPRi crRNA 1S	AAACtttgtttctttaatgtagatG	Spacer sequence for *coa* type1
Coa CRISPRi crRNA 1AS	AAAACatctacattaaagaaacaaa	Spacer sequence for *coa* type1
Coa CRISPRi crRNA 2S	AAACtagattgggcaattacatttG	Spacer sequence for *coa* type2
Coa CRISPRi crRNA 2AS	AAAACaaatgtaattgcccaatcta	Spacer sequence for *coa* type2
Coa CRISPRi crRNA 3S	AAACattgggcaattacattttggG	Spacer sequence for *coa* type3
Coa CRISPRi crRNA 3AS	AAAACccaaaatgtaattgcccaat	Spacer sequence for *coa* type3
BlaZ CRISPRi crRNA 1S	AAACaaaaattacaactgtaatatG	Spacer sequence for *blaZ* type1
BlaZ CRISPRi crRNA 1AS	AAAACatattacagttgtaattttt	Spacer sequence for *blaZ* type1
BlaZ CRISPRi crRNA 2S	AAACattacaactgtaatatcggaG	Spacer sequence for *blaZ* type2
BlaZ CRISPRi crRNA 2AS	AAAACtccgatattacagttgtaat	Spacer sequence for *blaZ* type2
Cas9 RT primer S	acgccaattggttgaaactc	qPCR for *dcas9* (Annealing: 60°C, Product size 225bp)
Cas9 RT primer AS	acgacggcatttagatacgc	qPCR for *dcas9* (Annealing: 60°C, Product size 225bp)
gyrB RT S	aggtcttggagaaatgaatg	qPCR for *gyrB* (Annealing: 62°C, Product size: 113bp)
gyrB RT AS	caaatgtttggtccgcttt	qPCR for *gyrB* (Annealing: 62°C, Product size: 113bp)
gap RT S	cggttacactgaagacgaa	qPCR for *gap* (Annealing: 57°C, Product size: 137bp)
gap RT AS	cgttatcataccaagctgc	qPCR for *gap* (Annealing: 57°C, Product size: 137bp)
femB RT S	tatcgtgccatttgaaggtc	qPCR for *femB* (Annealing: 62°C, Product size: 134bp)
femB RT AS	ggtttaatacgcccatccat	qPCR for *femB* (Annealing: 62°C, Product size: 134bp)
icaA RT S	agttgtcgacgttggctac	qPCR for *icaA* (Annealing: 60°C, Product size: 148bp)
icaA RT AS	ccaaagacctcccaatgt	qPCR for *icaA* (Annealing: 60°C, Product size: 148bp)
spa RT S	cggcactactgctgacaaaa	qPCR for *spa* (Annealing: 60°C, Product size: 117bp)
spa RT AS	ttagcatctgcatggtttgc	qPCR for *spa* (Annealing: 60°C, Product size: 117bp)
sec RT S	caaaacatgaaggaaaccac	qPCR for *sec* (Annealing: 60°C, Product size: 262bp)
sec RT AS	gcaggcatcatatcatacca	qPCR for *sec* (Annealing: 60°C, Product size: 262bp)
coa qPCR new primer S	aaaggctgacgaatcttgga	qPCR for *coa* (Annealing: 60°C, Product size: 178bp)
coa qPCR new primer AS	tgccactggttgtgttgttt	qPCR for *coa* (Annealing: 60°C, Product size: 178bp)
BlaZ realtime S	gatacttcaacgcctgctgc	qPCR for *blaZ* (Annealing: 60°C, Product size: 322bp)
BlaZ realtime AS	cactcttggcggtttcactt	qPCR for *blaZ* (Annealing: 60°C, Product size: 322bp)

Capital letters indicate restriction enzymes recognition sites or adaptor sequences

Table D in S1 file. Plasmid map of pBACi

No.	Start	Stop	Length	Direction	Function
1	414	449	36	-	Repeat (gttttagagctatgctgttttgaatggtcccaaaac)
2	450	455	6	-	BsaI recognition site (GGTCTC)
3	473	478	6	+	BsaI recognition site (GGTCTC)
4	480	515	36	-	Repeat (gttttagagctatgctgttttgaatggtcccaaaac)
5	668	4774	4107	-	dCas9
6	4985	5155	171	+	tracrRNA
7	5812	6426	615		Replication for *E. coli*
8	6985	7635	651	-	Chloramphenicol acetyltransferase
9	7914	8918	1005	-	Replication for *S. aureus*

Table E in S1 file. Detailed information of Silencing genes in this study

Genes	Proteins	Gene-loci	Protein-loci	Functions
spa	Staphylococcal protein A	Chromosome	Cell surface	IgG (Fc) binding/Immune-evasion
icaA	Intercellular adhesin protein A	Chromosome	Intra cellular*	PIA synthesis/biofilm formation*
sec	Staphylococcal enterotoxin C	SaPI**	Extra cellular	Causative agent of SFP and TSS***
coa	Coagulase	Chromosome	Extra cellular	Coagulation of fibrin
blaZ	β-lactamase	Plasmid	Extra cellular	Antibiotics resistance

*The final product, PIA, is present on the surface of bacteria, contributing to formation of biofilm.

***Staphylococcus aureus* pathogenicity island, mobile genetic element on *Staphylococcus* genome.

***Staphylococcal food poisoning and Toxic shock syndrome, the toxic mediated diseases caused by *S. aureus.*

Table F in S1 file. Putative RepB acceptable Gram-positive bacteria species other than *S. aureus*

Bacteria*	Ref.
Staphylococcus epidermidis	60
Staphylococcus saprophyticus	61
Staphylococcus cohnii	62
Bacillus cereus	Unpublished data (Accession no. U32369)
Bacillus subtilis	63, 64
Enterococcus faecium	65, 66
Enterococcus faecalis	63
Lactobacillus casei	67
Listeria innocua	68
Listeria monocytogenes	63
Streptococcus equinus	Unpublished data (ATCC 700338, EFM26591)
Streptococcus pasteurianus	Unpublished data (WP_041972885)
Ruminiclostridium thermocellum	Unpublished data (NC_021652)
Bacillus cereus	Unpublished data (PPU32369)
Staphylococcus sciuri	69
Staphylococcus hominis	Unpublished data (FPJS01000039)
Staphylococcus arlettae	Unpublished data (NC_016054)
Staphylococcus hyicus	Unpublished data (NC_016139)

*Similar proteins were found in NCBI (http://www.ncbi.nlm.nih.gov/)

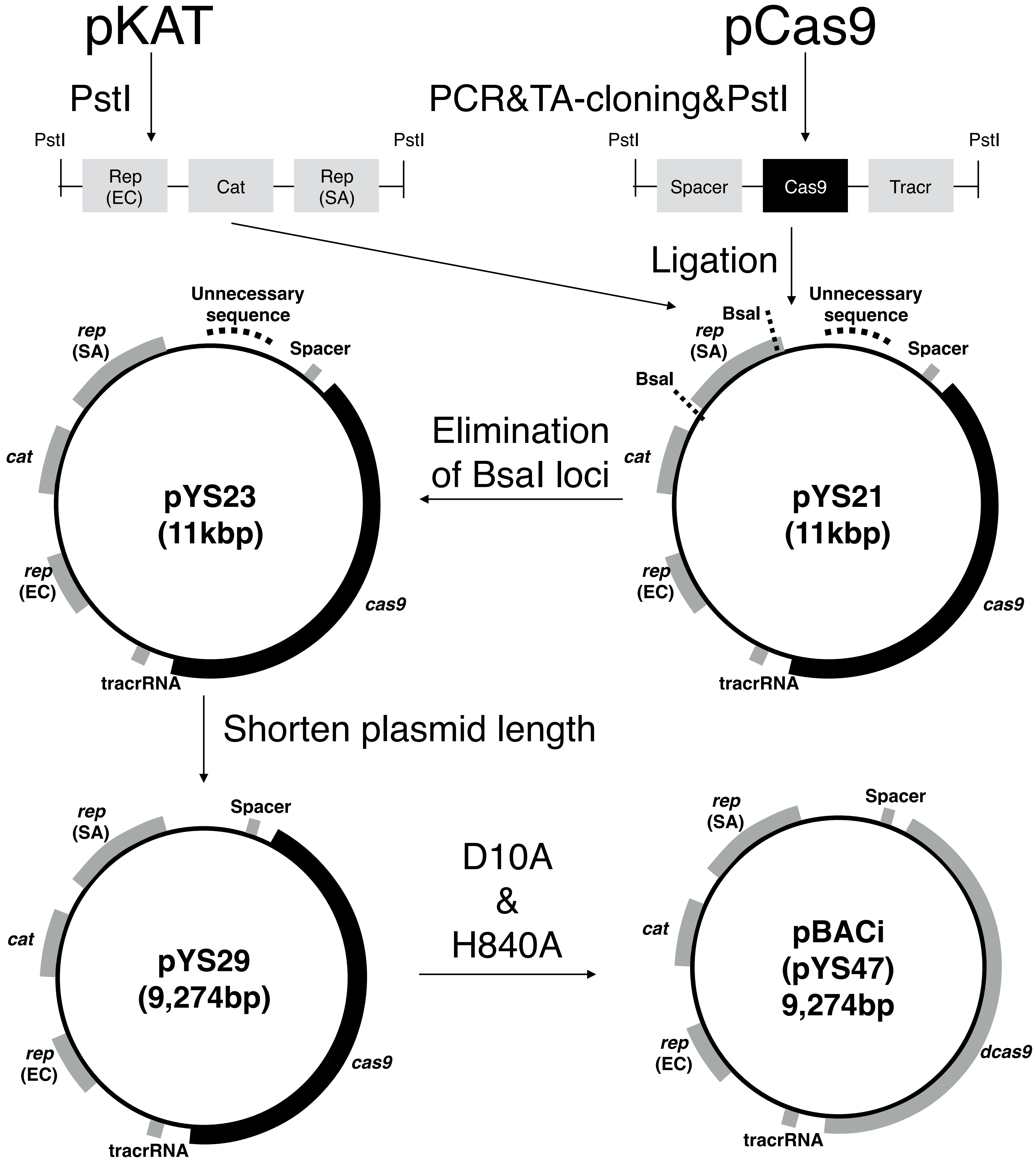

Fig A in S1 file. CRISPRi plasmid for *S. aureus*. Preparation of the CRISPRi *S. aureus-E. coli* shuttle vector is shown. Each operation was as follows. PCR&TA-cloning: Amplification of necessary genes involved in the CRISPR/Cas9 system and cloning of the DNA fragment with pGEM-T easy, PstI: PstI digestion; Elimination of BsaI loci: Deletion of two BsaI recognition sites (GGTCTC) within the vector, Shorten plasmid length: Deletion of unwanted sequence within vectors to reduce plasmid size and to improve electroporation efficiency, D10A&H840A: Change Cas9 to dCas9 with a two-amino acid mutation in two nuclease activity domains. *rep* (EC), the essential region for replication in *E. coli; cat,* chloramphenicol acetyltransferase gene; *repB* (SA), Replication initiator for *S. aureus;* Spacer, Nucleotide sequence involving repeats and BsaI recognition sites; *cas9,* Cas9 gene; tracrRNA, trans-activated crRNA coding region; *dcas*9, dead Cas9 gene; BsaI, BsaI recognition site.

Design of two primers containing spacer sequence on upstream of target gene

Primer S: 5'-AAACNNNNNNNNNNNNNNNNNNNNG-3'
Primer AS: 5'-AAAAC**NNNNNNNNNNNNNNNNNNNN**-3' *

Conduct kination of primers with PNK (NEB) in 50μl reaction mixture. After kination, add 2.5μl 1.5M NaCl and incubate 95°C for 5min with heat block. After heating, turn off heat block and incubated over 2 hours.

Digest pBACi with BsaI (NEB) and conduct gel extraction.

Ligation with purified digested plasmid and annealing spacer sequence mold using Ligation high Ver. 2 (TOYOBO) 4°C, o/n.

Transformation to DH5α. Conduct colony PCR and direct sequence with guide check S and AS primers

Extract successful plasmid and transformation to B strains (BL21 or BL21(DE3))

Extract plasmid from B strain and transform to *S. aureus* clinical isolates

Conduct PCR using Cas9 RT S-AS (or Guide check S-AS) to confirm successful transformation

Analyze phenotype changes of *S. aureus*

Fig B in S1 file. Protocol of CRISPRi for *S. aureus*

*: Bold letters are the complement sequence adjacent to PAM (NGG).

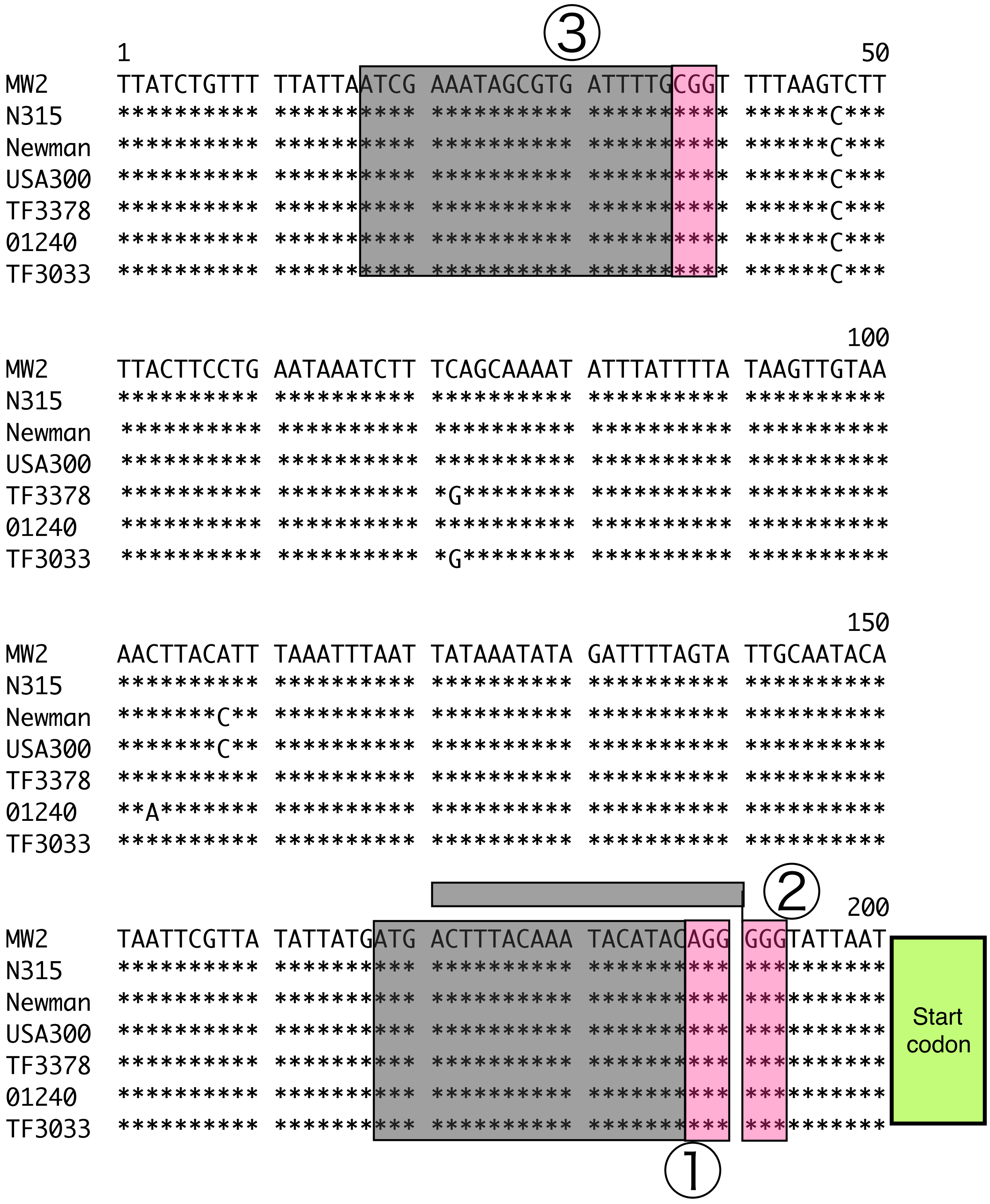

Fig C in S1 file. Alignment of nucleotide sequence around *spa*. The 200-bp sequences upstream of *spa* in seven strains are compared. Gray boxes, spacer sequences (1-3 corresponding to pYS103-105, respectively); red boxes, PAM sequence (NGG); MW2, BA000033; N315, BA000018; Newman, AP009351; USA300, NC_007793; The sequences of TF3378, 01240 and TF3033 were confirmed in this study.

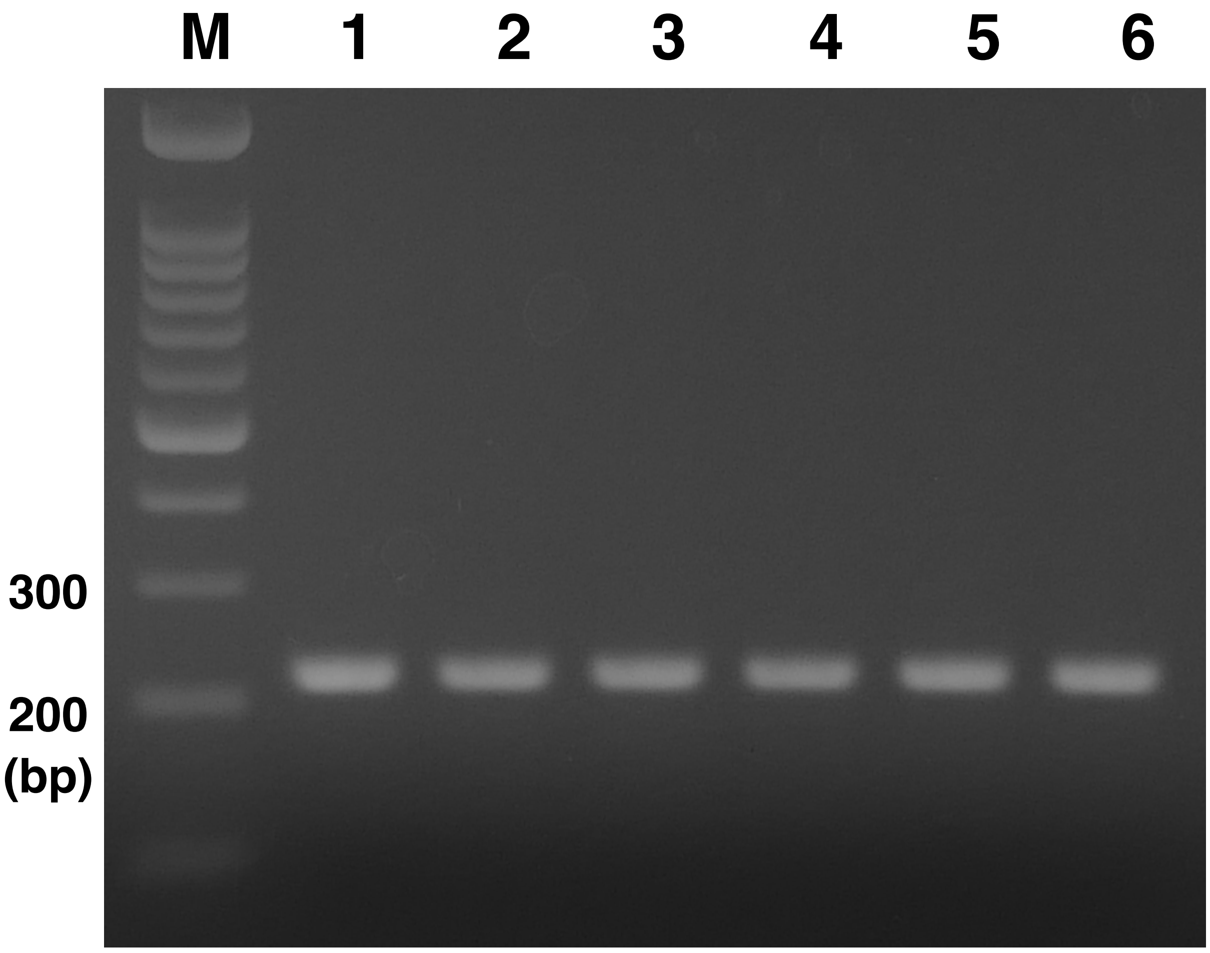

Fig D in S1 file. Successful transformation of pBACi. The pBACi-specific band was detected with PCR. The PCR protocol was the same as that in Figure 2B. 1, N315/pBACi; 2, Newman/pBACi; 3, USA300/pBACi; 4, TF3378/pBACi; 5, 01240/pBACi; 6, TF3033/pBACi

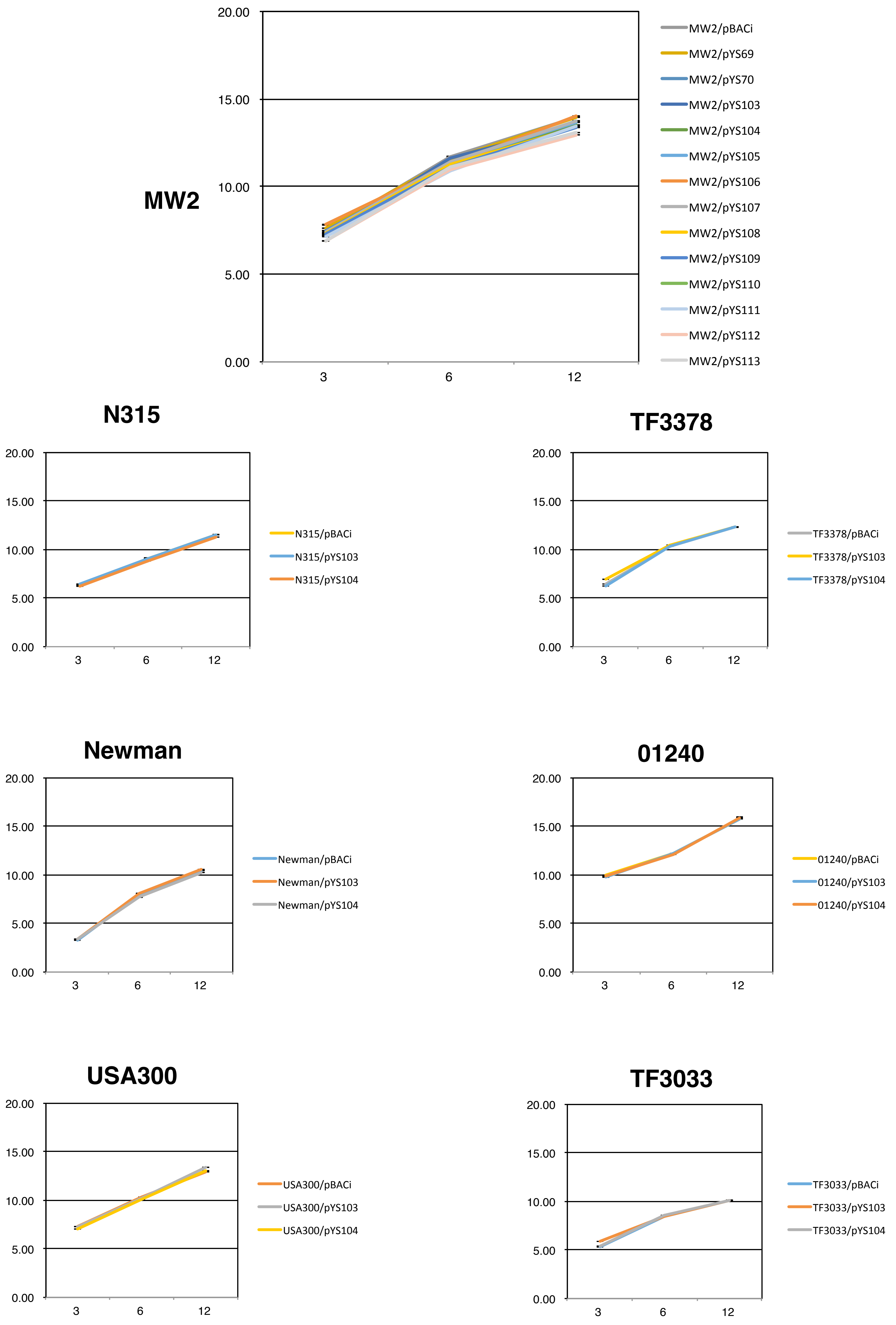

Fig E in S1 file. Growth curve of mutants. After inoculation of 1/100 volume o/n pre-culture media into fresh media (OD_{660}: ~0.1), temporal samples (3 h, 6 h and 12 h) were taken. The cultured media were diluted 10-fold and measured with SPECTRONIC 200. Three independent assays were performed. Average and standard error (SE) are shown.

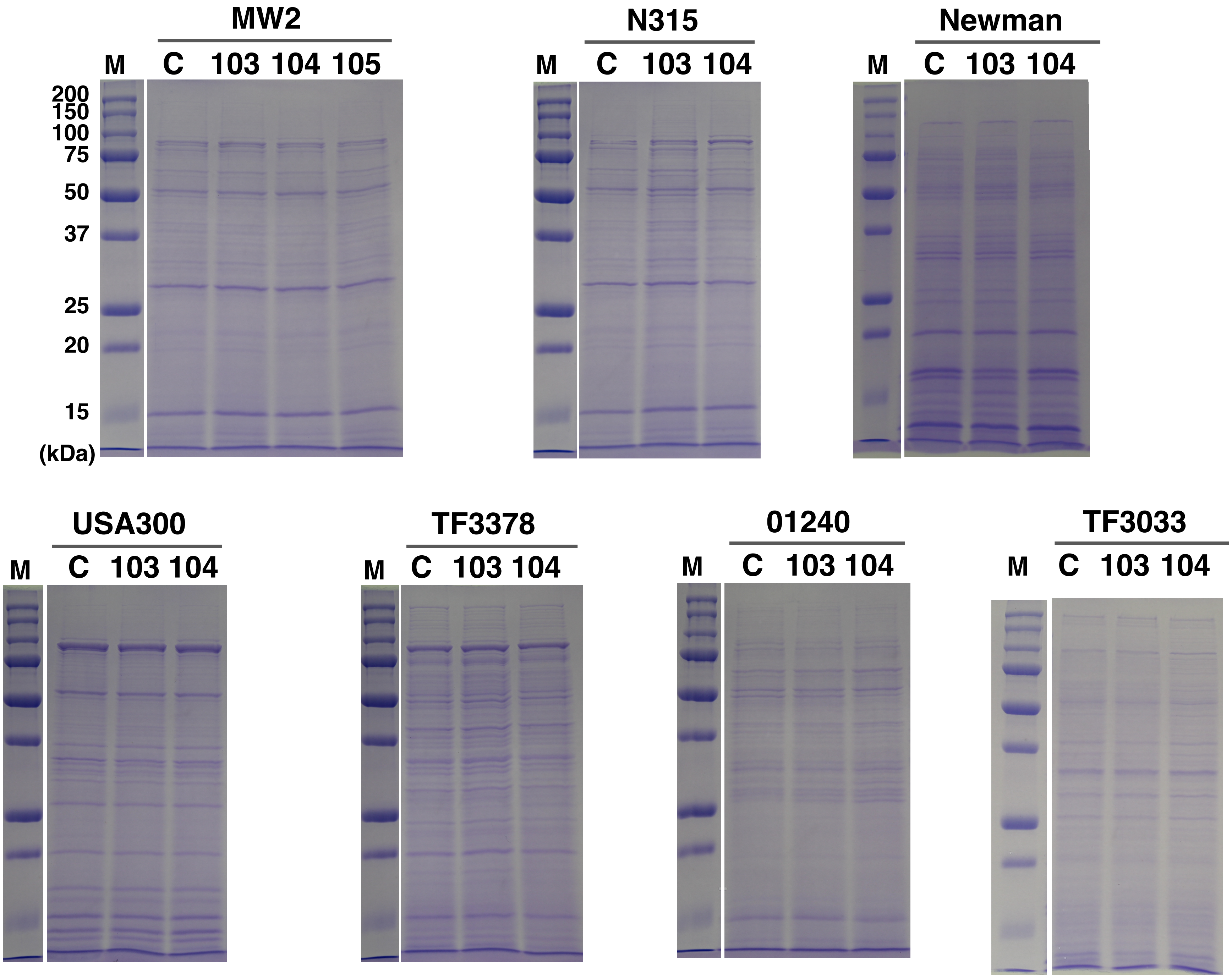

Fig F in S1 file. SDS-PAGE result for each strain. The samples for western blotting were subjected to SDS-PAGE and CBB staining. pBACi (C), Vector control; pYS103 (103), spaA silenced knockdown vector 1; pYS104 (104), spaA silenced knockdown vector 2; pYS105 (105), spaA silenced knockdown vector 3; M, Molecular marker.

Supplemental reference

53. Baba T. et al. (2002) Genome and virulence determinants of high virulence community-acquired MRSA. *Lancet.* **359**, 1819-1827.

54. Kuroda M. et al. (2001) Whole genome sequencing of meticillin-resistant *Staphylococcus aureus*. *Lancet*. **357**, 1225-1240.

55. Baba T., Bae T., Schneewind O., Takeuchi F. & Hiramatsu K. (2008) Genome sequence of *Staphylococcus aureus* strain Newman and comparative analysis of staphylococcal genomes: polymorphism and evolution of two major pathogenicity islands. *J. Bacteriol.* **190**, 300-310.

56. Diep B.A. et al. (2006) Complete genome sequence of USA300, an epidemic clone of community-acquired meticillin-resistant *Staphylococcus aureus*. *Lancet* 367, 731-739.

57. Traber K. & Novick R. (2006) A slipped-mispairing mutation in AgrA of laboratory strains and clinical isolates results in delayed activation of agr and failure to translate delta- and alpha-haemolysins. *Mol Microbiol.* **59**, 1519-1530.

58. Kato F., Nakamura M., Sugai M. (2017) The development of fluorescent protein tracing vectors for multicolor imaging of clinically isolated *Staphylococcus aureus*. *Sci. Rep.* **7**, 2865.

59. Kato F. & Sugai M. (2011) A simple method of markerless gene deletion in *Staphylococcus aureus*. *J Microbiol Methods.* **87**, 76-81.

60. Conlan S. et al. (2012) *Staphylococcus epidermidis* pan-genome sequence analysis reveals diversity of skin commensal and hospital infection-associated isolates. *Genome Biol.* **13**, R64.

61. Hauschild T., Lüthje P. & Schwarz S. (2006) Characterization of a novel type of MLSB resistance plasmid from *Staphylococcus saprophyticus* carrying a constitutively expressed *erm*(C) gene. *Vet. Microbiol.* **115**, 258-263.

62. Allignet J., Liassine N., el Solh N. (1998) Characterization of a staphylococcal plasmid related to pUB110 and carrying two novel genes, *vatC* and *vgbB*, encoding resistance to streptogramins A and B and similar antibiotics. *Antimicrob. Agents Chemother.* **42**, 1794-1798.

63. Charpentier E., Gerbaud G. & Courvalin P. (1999) Conjugative mobilization of the rolling-circle plasmid pIP823 from *Listeria monocytogenes* BM4293 among gram-positive and gram-negative bacteria. *J. Bacteriol.* **181**, 3368-3374.

64. Diderichsen B., Poulsen GB. & Jørgensen S.T. (1993) A useful cloning vector for *Bacillus subtilis*. *Plasmid.* **30**, 312-315.

65. Qin X. et al. (2012) Complete genome sequence of *Enterococcus faecium* strain TX16 and comparative genomic analysis of *Enterococcus faecium* genomes. *BMC Microbiol.* **12**, 135.

66. van Schaik W. et al. (2010) Pyrosequencing-based comparative genome analysis of the nosocomial pathogen *Enterococcus faecium* and identification of a large transferable pathogenicity island. *BMC Genomics.* **11**, 239.

67. Shimizu-Kadota M., Shibahara-Sone H. & Ishiwa H. (1991) Shuttle plasmid vectors for *Lactobacillus casei* and *Escherichia coli* with a minus origin. *Appl. Environ. Microbiol.* **57**, 3292-3300.

68. Bertsch D., Anderegg J., Lacroix C., Meile L. & Stevens M.J. (2013) pDB2011, a 7.6 kb multidrug resistance plasmid from *Listeria innocua* replicating in Gram-positive and Gram-negative hosts. *Plasmid.* **70**, 284-287.

69. Zeman M., Mašlaňová I., Indráková A., Šiborová M., Mikulášek K., Bendíčková K., Plevka P., Vrbovská V., Zdráhal Z., Doškař J. & Pantůček R. (2017) *Staphylococcus sciuri* bacteriophages double-convert for staphylokinase and phospholipase, mediate interspecies plasmid transduction, and package *mecA* gene. *Sci. Rep.* **7**, 46319.
